# Correction: Yi et al. An Innovative Inducer of Platelet Production, Isochlorogenic Acid A, Is Uncovered through the Application of Deep Neural Networks. *Biomolecules* 2024, *14*, 267

**DOI:** 10.3390/biom14060655

**Published:** 2024-06-04

**Authors:** Taian Yi, Jiesi Luo, Ruixue Liao, Long Wang, Anguo Wu, Yueyue Li, Ling Zhou, Chengyang Ni, Kai Wang, Xiaoqin Tang, Wenjun Zou, Jianming Wu

**Affiliations:** 1 State Key Laboratory of Southwestern Chinese Medicine Resources, School of Pharmacy, Chengdu University of Traditional Chinese Medicine, Chengdu 611137, China; yitaian@stu.cdutcm.edu.cn (T.Y.); liyueyue@stu.cdutcm.edu.cn (Y.L.); 2 Department of Chemistry, School of Basic Medical Sciences, Southwest Medical University, Luzhou 646000, China; ljs@swmu.edu.cn; 3 Department of Pharmacology, School of Pharmacy, Southwest Medical University, Luzhou 646000, Chinawanglong1226@swmu.edu.cn (L.W.); wuanguo@swmu.edu.cn (A.W.); zhouling@stu.swmu.edu.cn (L.Z.); nichengyang@stu.swmu.edu.cn (C.N.); 20220599120055@stu.swmu.edu.cn (K.W.); 20210599120093@stu.swmu.edu.cn (X.T.); 4 The Institute of Cardiovascular Research, Key Laboratory of Medical Electrophysiology of Ministry of Education, Luzhou 646000, China

**Error in Figure**

In the original publication [1], there was a mistake in Figure 5 as published. The ICGA-A (10 μM) group of K562 cells in Figure 5A was incorrectly placed. The corrected Figure 5 appears below. 

The authors state that the scientific conclusions are unaffected. This correction was approved by the Academic Editor. The original publication has also been updated.

## Figures and Tables

**Figure 5:**
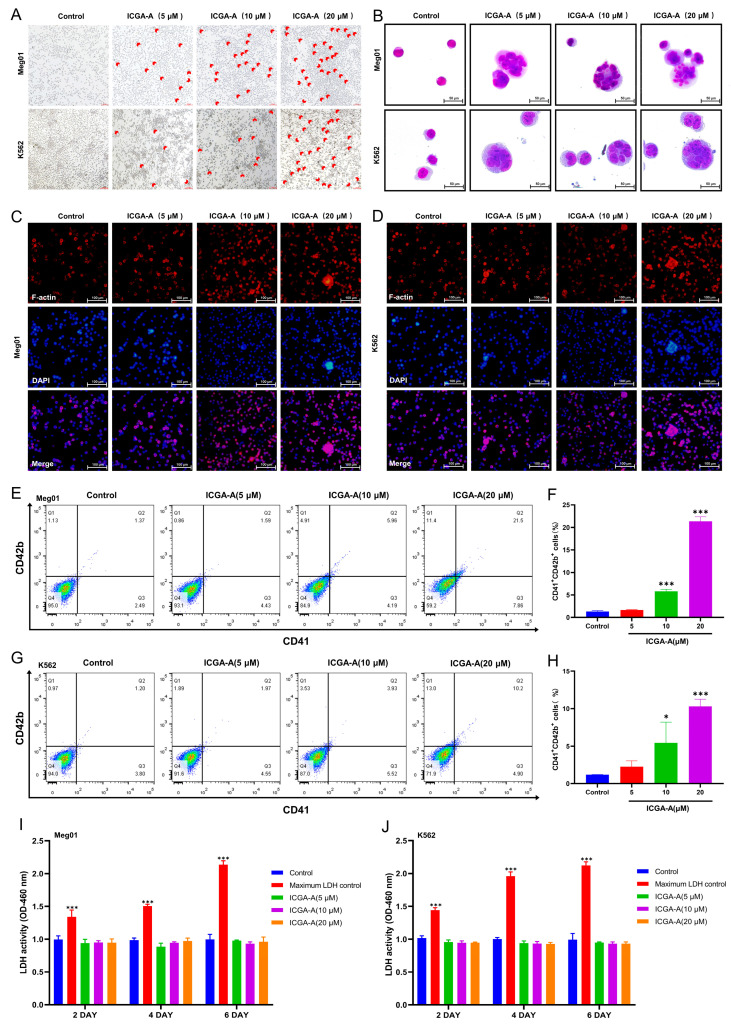
ICGA-A induces MK differentiation of Meg-01 and K562 cells. (**A**) Microscope photographs of Meg-01 and K562 cells treated with or without ICGA-A (5, 10 and 20 μM) for 5 days. Scale bar: 100 µm. (**B**) Giemsa staining of Meg-01 and K562 cells with or without ICGA-A (5, 10 and 20 μM) treatment for 5 days. Scale bar: 50 µm. (**C**,**D**) Phalloidin staining of Meg-01 and K562 cells with or without ICGA-A (5, 10 and 20 μM) treatment for 5 days. Scale bar: 100 μm. (**E**,**G**) CD41 and CD42b expression on Meg-01 and K562 cells with or without ICGA-A (5, 10 and 20 μM) treatment for 5 days. (**F**,**H**) The proportion of CD41+CD42b+ cells of Meg-01 and K562 cells in control- and ICGA-A-treated groups. Data are presented as mean ± SD (n = 3). (**I**,**J**) Detection of LDH activity of Meg-01 and K562 cells after treatment with or without ICGA-A (5, 10 and 20 μM) for 2, 4 and 6 days. Maximum LDH control represents the total amount of LDH present in the cells. Data are presented as mean ± SD (n = 3, ANOVA). * *p* < 0.05, *** *p* < 0.001 vs. the control group.
