# Supplementary material for: An Innovative Inducer of Platelet Production, Isochlorogenic Acid A, Is Uncovered through the Application of Deep Neural Networks
Source: Biomolecules. 2024 Feb 23;14(3):267. doi: 10.3390/biom14030267 (PMC10968240; doi:10.3390/biom14030267)
Supplement: Supplementary file 1 [file biomolecules-14-00267-s001.zip › TableS1.pptx]

## Slide 1
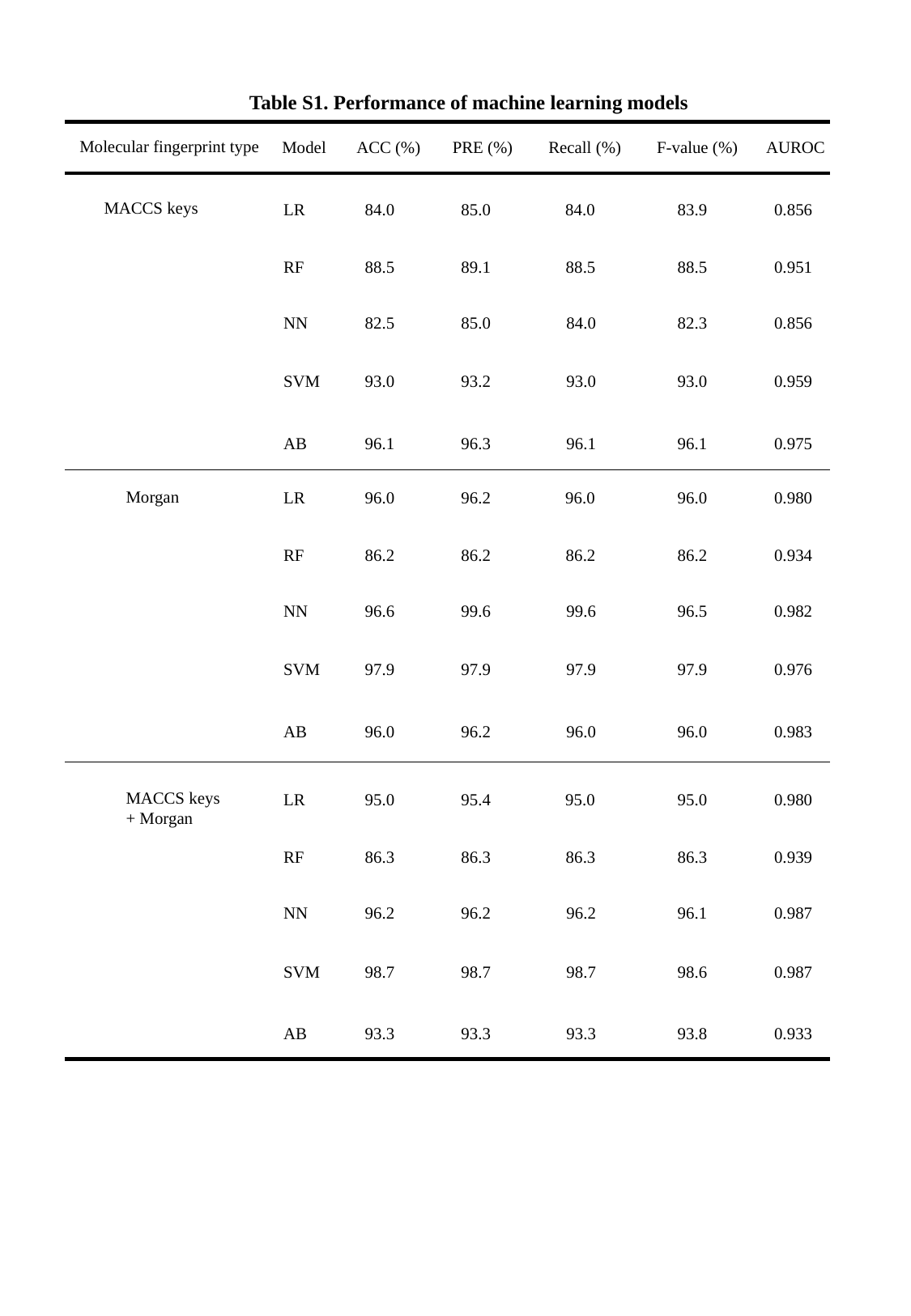

Table S1. Performance of machine learning models
Model
ACC (%)
PRE (%)
Recall (%)
F-value (%)
AUROC
Molecular fingerprint type
MACCS keys
LR
84.0
85.0
84.0
83.9
0.856
RF
88.5
89.1
88.5
88.5
0.951
NN
82.5
85.0
84.0
82.3
0.856
SVM
93.0
93.2
93.0
93.0
0.959
AB
96.1
96.3
96.1
96.1
0.975
Morgan
LR
96.0
96.2
96.0
96.0
0.980
RF
86.2
86.2
86.2
86.2
0.934
NN
96.6
99.6
99.6
96.5
0.982
SVM
97.9
97.9
97.9
97.9
0.976
AB
96.0
96.2
96.0
96.0
0.983
MACCS keys
+ Morgan
LR
95.0
95.4
95.0
95.0
0.980
RF
86.3
86.3
86.3
86.3
0.939
NN
96.2
96.2
96.2
96.1
0.987
SVM
98.7
98.7
98.7
98.6
0.987
AB
93.3
93.3
93.3
93.8
0.933
