# Supplementary material for: An Innovative Inducer of Platelet Production, Isochlorogenic Acid A, Is Uncovered through the Application of Deep Neural Networks
Source: Biomolecules. 2024 Feb 23;14(3):267. doi: 10.3390/biom14030267 (PMC10968240; doi:10.3390/biom14030267)
Supplement: Supplementary file 1 [file biomolecules-14-00267-s001.zip › TableS2.pptx]

## Slide 1
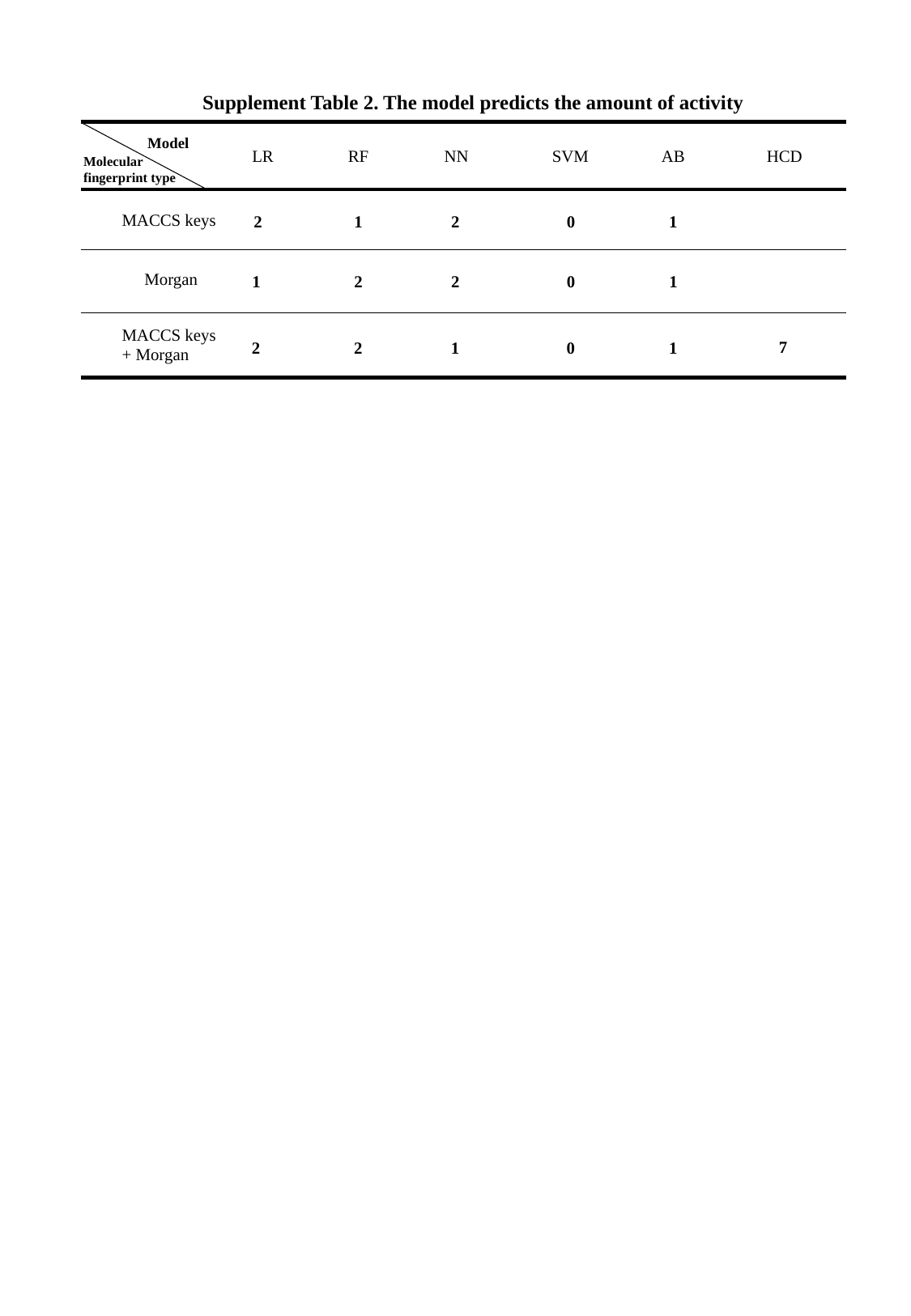

Supplement Table 2. The model predicts the amount of activity
Model
LR
NN
SVM
RF
AB
HCD
Molecular fingerprint type
MACCS keys
2
1
2
0
1
Morgan
1
2
2
0
1
MACCS keys
+ Morgan
7
2
2
1
0
1
