# Supplementary figures and images for: An Innovative Inducer of Platelet Production, Isochlorogenic Acid A, Is Uncovered through the Application of Deep Neural Networks
Source: Biomolecules. 2024 Feb 23;14(3):267. doi: 10.3390/biom14030267 (PMC10968240; doi:10.3390/biom14030267)

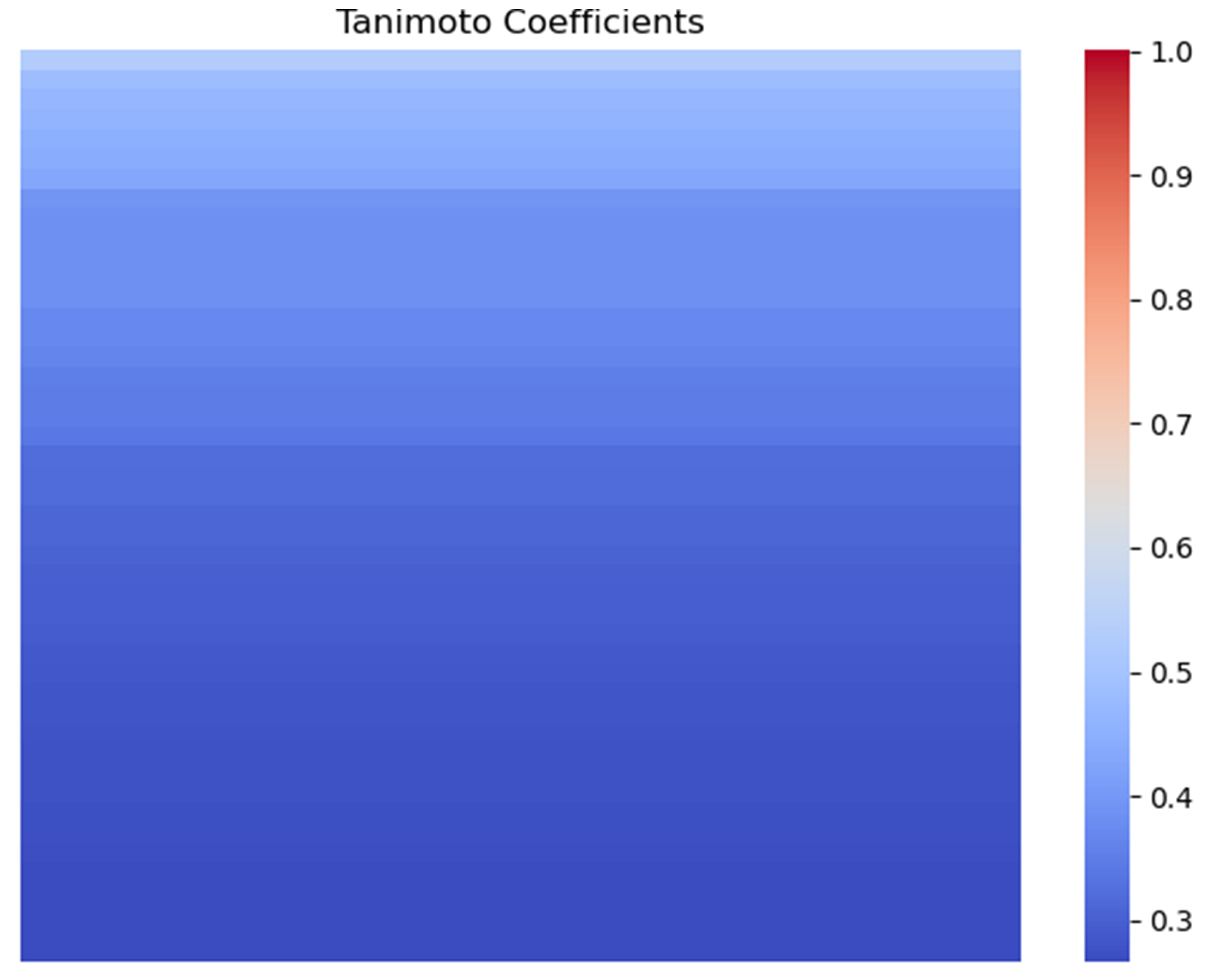

Supplement: Supplementary file 1 [file biomolecules-14-00267-s001.zip › FiguerS2.jpg]

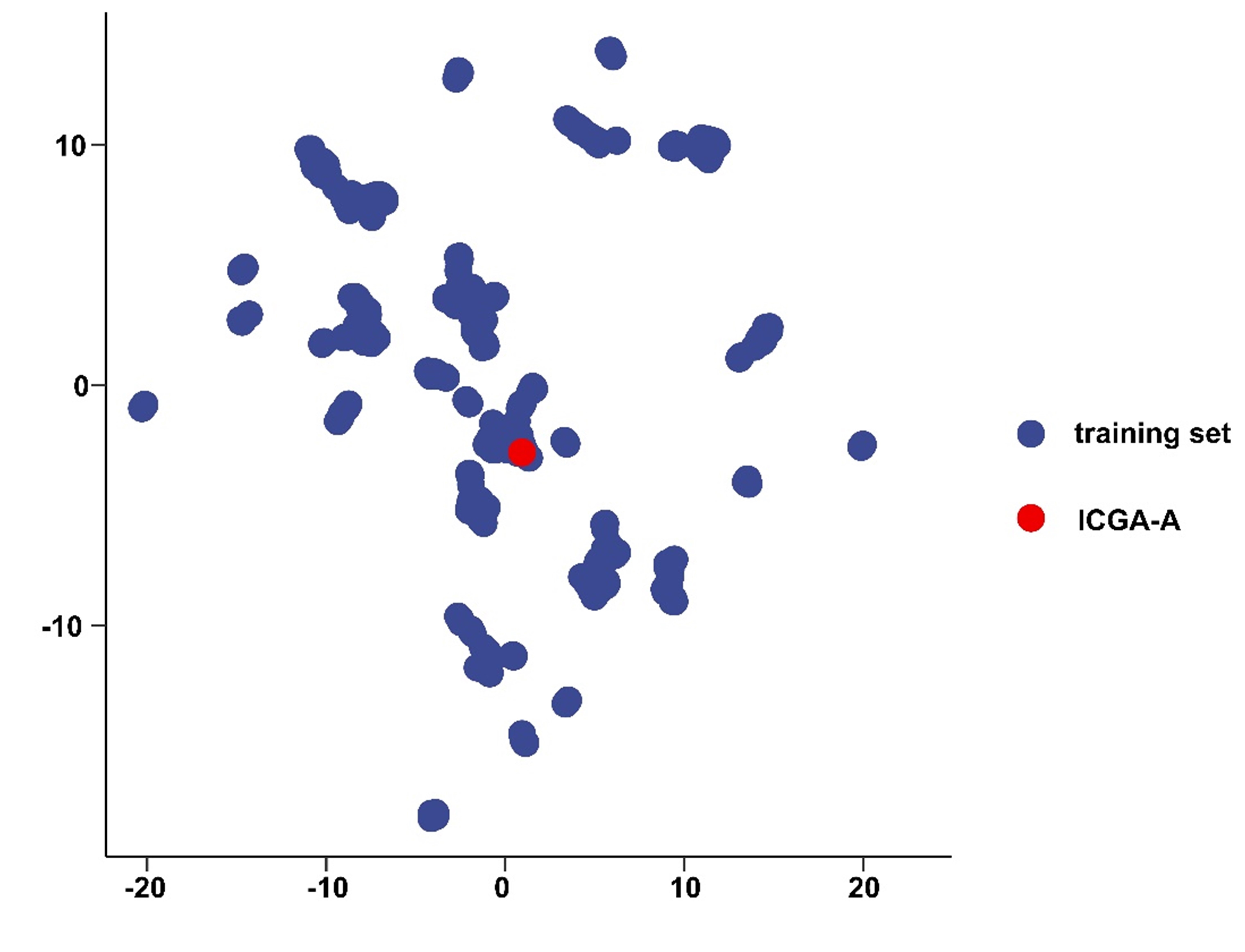

Supplement: Supplementary file 1 [file biomolecules-14-00267-s001.zip › FiguerS3.jpg]
